# Design and Simulation of Magnetic Shielding Structure Based on Closed-Loop TMR Current Sensor

**DOI:** 10.3390/mi16030272

**Published:** 2025-02-27

**Authors:** Qiuyang Li, Suqin Xiong, Shuo Wang, Xianguang Dong, Haifeng Zhang

**Affiliations:** 1 China Electric Power Research Institute Co., Ltd., Beijing 100192, China; liqiuyang@epri.sgcc.com.cn (Q.L.); xiongsq@epri.sgcc.com.cn (S.X.); 2 MEMS Center, Harbin Institute of Technology, Harbin 150001, China; zhanghf@hit.edu.cn; 3 State Grid Shandong Electric Power Company Marketing Service Center (Metrology Center), Jinan 250001, China; dongxianguang@sd.sgcc.com.cn

**Keywords:** current sensor, closed-loop TMR current sensor, COMSOL Multiphysics, shielded structure

## Abstract

With the rapid development of current sensor technology, tunnel magnetoresistance (TMR) current sensors have been widely adopted in industrial detection due to their high sensitivity, excellent linearity, and broad measurement range. This study focuses on closed-loop TMR current sensors, utilizing COMSOL Multiphysics 6.2 software and the finite element method to conduct an in-depth analysis of structural parameters affecting sensor sensitivity. A novel magnetic shielding package architecture is proposed and designed. Simulation results demonstrate that the shielding efficiency of this structure improves by 44.3% compared to a single magnetic ring under a stray magnetic field of 0.1 mT along the sensing axis. At the same time, the measurement accuracy is 2.1 times higher than that of traditional structures. Current detection experiments conducted in a strong magnetic field environment further validate that the shielding package effectively suppresses external electromagnetic interference, significantly enhancing sensor stability and measurement accuracy. This research provides important theoretical and practical insights for applying high-precision TMR current sensors in complex electromagnetic environments.

## 1. Introduction

### 1.1. Research Background

The evolving power grid industry demands current sensors with enhanced accuracy, extensive measurement range, and high-frequency broadband capabilities. Conventional devices like Rogowski coils are limited by their inability to measure DC currents, susceptibility to saturation under large fault currents, and bulky design due to iron cores. Similarly, Hall current sensors face challenges such as low sensitivity, high power consumption, poor linearity, and significant temperature dependence. These current sensors are difficult to meet the new development needs of the new generation of power system online detection and high-precision fault diagnosis. Tunnel magnetoresistance (TMR) current sensors are gradually being applied to power monitoring and relay protection due to their noncontact structure, high sensitivity, and low power consumption [1,2]. However, in practical applications, the accuracy of TMR current sensors is easily affected by temperature, external magnetic field interference, and other factors. Therefore, it is necessary to study the structure of TMR current sensors. It is necessary to ensure that the TMR current sensor has high sensitivity and to improve its anti-interference ability by designing appropriate structures and shielding methods. This study focuses on the structural design and magnetic shielding optimization of closed-loop TMR current sensors.

### 1.2. Related Research Work

In recent years, extensive research has been conducted to improve the performance of TMR current sensors. Literature [3] introduces a closed-loop current sensor utilizing zero magnetic flux technology to eliminate temperature-induced output drift. Temperature drift refers to the deviation of the sensor output signal caused by changes in ambient temperature, which significantly reduces measurement accuracy and stability. Weiss et al. [4] designed a magnetic field sensor array utilizing the closed-loop structure principle, further demonstrating the potential of closed-loop structures in enhancing sensor performance. Additionally, literature [5] introduced an electromagnetic interference compensation method based on dual TMR chips, which significantly improved the sensor’s anti-interference capability by separately measuring the synthetic magnetic field and interference magnetic field signals. However, existing research still has some limitations: TMR current sensors with array structures exhibit insufficient sensitivity and anti-interference capability, while open-loop structures are prone to temperature drift. This article is based on a TMR current sensor with a closed-loop structure and studies the influence of magnetic ring structure parameters on its sensitivity. A practical magnetic shielding structure is designed to reduce the measurement error of the current sensor in the geomagnetic field and external radiation interference.

### 1.3. Major Contributions

To overcome the above limitations, in this paper, the structure of a TMR current sensor using the magnetic ring and shielding shell is presented as a current sensor system. The influence of the structural parameters of the magnetic ring on the sensitivity is also simulated, and a new magnetic shield structure is designed. This structure has the following main advantages:

1. The system adopts a double-layer shielding structure, and selects the material with high permeability, and the shielding efficiency is very good.
2. The shielding structure can be loaded with an external circuit board to protect the internal circuit.
3. The magnetic loop structure can effectively increase the sensitivity of current measurement and play a certain role in magnetic shielding.

### 1.4. Article Organization

The rest of this article is organized as follows: In Section 2, we analyze the structural parameters of the TMR current sensor and carry out finite element simulations to obtain the influence of different structural parameters on the performance of the sensor. In Section 3, we analyze the principle of magnetic shielding and analyze the influence of the thickness of the shielding shell and the number of layers on the shielding efficiency for the annular structure of the TMR current sensor and design a new shielding structure, which not only has good shielding effectiveness but also can protect the internal circuit. In Section 4, we conduct a simulation study and practical tests on the shielded housing, and the test shows that the measurement error of the shielded TMR current sensor is much smaller than that of the unshielded TMR current sensor. Finally, the discussion and conclusions are provided in Section 5.

## 2. Structural Design and Simulation of Closed-Loop TMR Current Sensor

### 2.1. Principles of TMR Effect and MTJ Structure

**Figure 1:**
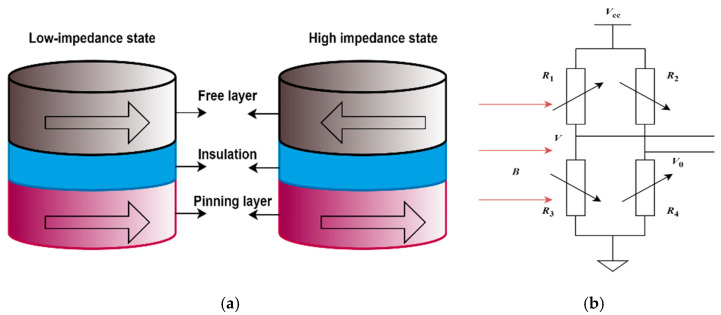
(**a**) Typical structure of MTJ sensor unit; (**b**) the schematic of the bridge circuit and resistor structure.

The tunneling magnetoresistance effect refers to the phenomenon found in the three-layer structure of ferromagnetic film material/intermediate insulation layer/ferromagnetic film material, in which the tunneling resistance changes with the relative orientation of the magnetic moments of the two ferromagnetic layers, and its mechanism is the spin-dependent tunneling effect. Since the discovery of the tunneling magnetoresistance effect, people have studied various tunnel junction structures from both theoretical and experimental aspects. Generally, the ferromagnetic/insulator/ferromagnetic three-layer film structure is called a magnetic tunnel junction (MTJ). The typical structure of the MTJ sensor unit is shown in Figure 1a, including the free layer, insulation, and pinning layer.

The resistance and tunneling current of the MTJ sensing unit are determined by the relative orientation of the magnetization of the free layer and the pinned layer. When the external magnetic field of the MTJ sensor unit increases, the magnetization direction of the magnetic free layer changes, while the magnetization direction of the ferromagnetic pinning layer remains unchanged. At this time, the relative direction of the magnetization of the free layer and the pinning layer changes, and the tunnel resistance will change greatly.

The TMR element comprises four tunneling magnetoresistors with identical resistance, each varying in response to an external magnetic field along the same sensitivity direction on the diagonal bridge arm. Figure 1b illustrates the schematic of the bridge circuit and resistor structure [6,7,8]. *R*_1_, *R*_2_, *R*_3_, and *R*_4_ are TMR magnetoresistive elements with four different sensitive directions. The sensitive directions of *R*_1_ and *R*_4_ are consistent and antiparallel to *R*_2_ and *R*_3_. The output differential voltage *u*_0_ is *V* − *V*_0._

The TMR chip translates variations in the external magnetic field into corresponding output voltage changes [9]. Within the linear range, the output voltage *u*_0_ of the TMR sensor chip is directly proportional to the magnetic induction intensity *B*:

(1) $$u_{0}=K_{0}\cdot B$$

*K*_0_ represents the sensitivity of the TMR sensor chip.

### 2.2. Principle of Closed-Loop TMR Current Sensor

A single TMR chip can sense that the magnetic field generated by the wire is very small, thus reducing the sensitivity. There may be errors in the measurement results of the sensor due to the wire offset.

**Figure 2:**
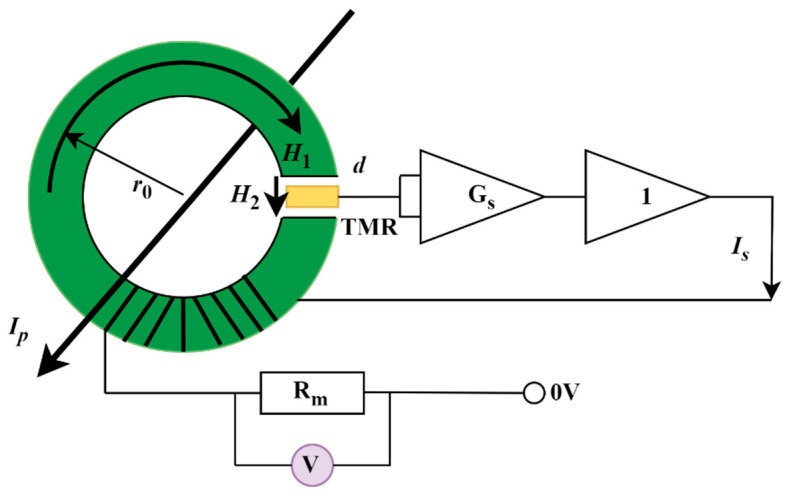
Structure of a closed-loop TMR current sensor.

To improve the sensitivity of the TMR current sensor, a concentrating ring composed of soft magnetic material was introduced. Figure 2 illustrates a toroidal core structure designed for current-carrying wires to traverse its interior. The magnetic ring not only enhances the sensitivity of the TMR current sensor by concentrating the magnetic field but also acts as a magnetic shield to reduce external magnetic interference. This dual function makes the magnetic ring a critical component in improving the sensor’s performance.

The TMR sensing chip is centrally positioned in the magnetic ring’s air gap, aligning its sensitive axis parallel to the magnetic field direction within the gap. By incorporating a feedback coil, open-loop TMR current sensors can be transformed into closed-loop TMR current sensors, enabling the TMR element to function at zero magnetic flux [3,10,11,12]. The design involves the wire passing through the magnetic ring, positioning the TMR element centrally within the air gap. The output signal of the TMR element is amplified by a differential op amp, and the amplified signal is amplified by the power to generate a feedback current. The feedback coil is wound on the magnetic ring, producing a magnetic field opposite to that of the measured wire, thereby reducing the magnetic field strength in the air gap to zero. Consequently, the magnetic field generated by the feedback coil aligns with that of the measured wire, allowing for the calculation of the feedback current.

*I_P_* is the current to be measured, *I_s_* is the feedback current, *G_S_* is the amplification circuit, 1 is the power amplification circuit, and *R_m_* is the feedback resistance.

Based on Ampere’s loop theorem, it is evident that:

(2) $$\oint H\cdot dl=H_{1}\cdot (2\pi r_{0}-d)+H_{2}\cdot d=I$$

*I* is the current through the wire, *H*_1_ is the magnetic field strength inside the magnetic ring, *H*_2_ is the magnetic field strength at the air gap, *r*_0_ is the average radius of the magnetic ring, and *d* is the air gap width. According to the magnetic flux continuity theorem, the magnetic induction intensity is consistent in both the core and the air gap:

(3) $$B=\mu_{r}H_{1}=\mu_{0}H_{2}$$

In this context, *µ_r_* indicates the relative permeability of the magnetic ring, whereas *µ*_0_ represents that of air.

*I* is the measured current, *H* is the magnetic field strength generated by this current in the magnetic ring’s air gap, *V_t_* is the TMR chip’s output voltage, and *V*_0_ is the system’s output voltage. *K_r_* is the electromagnetic conversion sensitivity of the magnetic ring, combined with the magnetic ring structure, which can be obtained from Ampere’s law.

(4) $$Kr=\frac{H}{I}=\frac{1}{d+\frac{\pi (R+r)-d}{\mu_{r}}}$$

**Figure 3:**
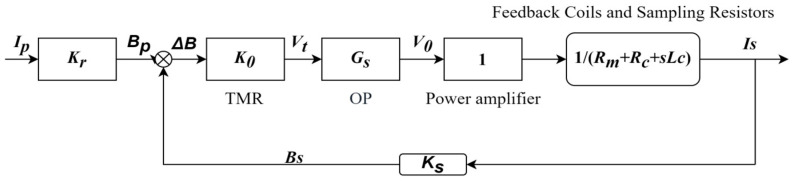
Closed-loop current sensor model.

The mathematical model of the TMR closed-loop current sensor based on the magnetic ring structure is shown in Figure 3.

In Figure 3, *I_P_* is the system input, *I_s_* is the system output, and *K*_0_ is the sensitivity of the TMR sensor chip. Because the output voltage of TMR components is easily affected by temperature, the value of *K*_0_ will vary with changes in external temperature. *B_P_* is the magnetic induction intensity component of the magnetic field generated by the primary current *I_P_* on the sensitive axis of the TMR sensor chip, *G_S_* is the gain of the instrument amplifier, 1/(*Rm* + *Rc* + *sLc*) is the reciprocal of the sampling resistance and the impedance of the feedback coil is the feedback current, *K_S_* is the feedback coefficient, and *B_S_* is the component of the magnetic induction intensity generated by is on the sensitive axis of the TMR sensor chip. *ΔB* is the difference between *B_P_* and *B_S_*. *V_t_* and *V*_0_ are the voltage output by the chip and the voltage output by the amplifier, respectively.

In this case, the relationship between *I_P_* and *I_s_* is as follows:

(5) $$Is(s)=\frac{K_{r}K_{0}G_{s}}{Z(s)-K_{0}G_{s}K_{s}}I_{p}(s)$$

(6) $$Z(s)=R_{m}+R_{c}+sLc$$

Based on the transfer function, the feedback current maintains a proportional relationship with the primary current, enhancing both the measurement accuracy and linearity of the closed-loop TMR current sensor.

TMR components are affected by the outside temperature, i.e., changes with temperature. However, when *K*_0_*G_s_* >> *Z*(*s*), the transfer function can be approximated as:

(7) $$H(s)=-\frac{Kr}{Ks}$$

From Formula (7), it can be seen that the output of the sensor has almost no relationship with *K*_0_, so the output of the sensor will not produce significant errors due to temperature changes, thus avoiding temperature drift. By combining the magnetic ring and the shielding structure designed later, the sensor not only avoids errors caused by temperature but also reduces errors caused by external geomagnetic interference. In the next section, we will conduct parameter simulations of the magnetic ring structure to study its influence on the sensor’s sensitivity and analyze the shielding effect of the magnetic ring. These simulations will provide valuable insights into optimizing the magnetic ring’s design for both sensitivity enhancement and external interference reduction.

### 2.3. Simulation of Magnetic Ring Structure Parameters and Feedback Coils

The closed-loop TMR current sensor consists of a magnetic ring and a feedback coil. The structural parameters of the magnetic ring influence its magnetic aggregation effect, thereby affecting the sensitivity of the TMR current sensor. Therefore, conducting simulations of the magnetic ring’s structural parameters to study the impact of different parameters on sensitivity is crucial for determining the magnetic ring’s structure and the subsequent design of the magnetic shielding enclosure.

**Figure 4:**
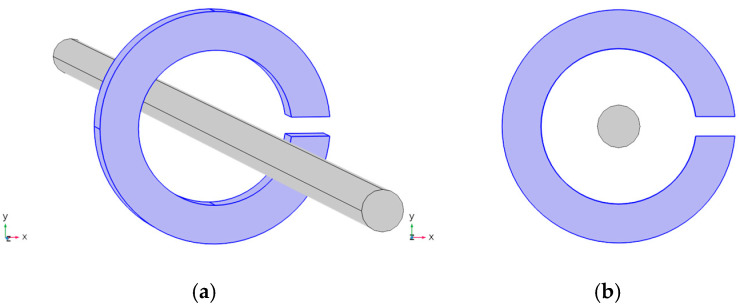
Schematic diagram of finite element simulation modeling: (**a**) 3D schematic diagram; (**b**) 2D schematic diagram of XY plane.

This study employs COMSOL Multiphysics 6.2 for finite element simulation, modeling a copper conductor with a radius of 5 mm and a maximum current of 100 A. The magnetic ring is composed of a soft magnetic material with a permeability of 20,000. To facilitate the simulation, the length of the conductor is set to 50 cm, and the current passing through it is 1 A. Figure 4 illustrates the modeling schematic in the COMSOL software. Specifically, Figure 4b shows a two-dimensional schematic in the xy-plane. In this simulation, the sensitivity of the sensor is characterized by the magnitude of the magnetic flux density at the center of the air gap. Since the sensitive axis of the TMR chip is set as the y-axis, the magnetic flux density obtained from the simulation results corresponds to its component along the y-axis.

**Figure 5:**
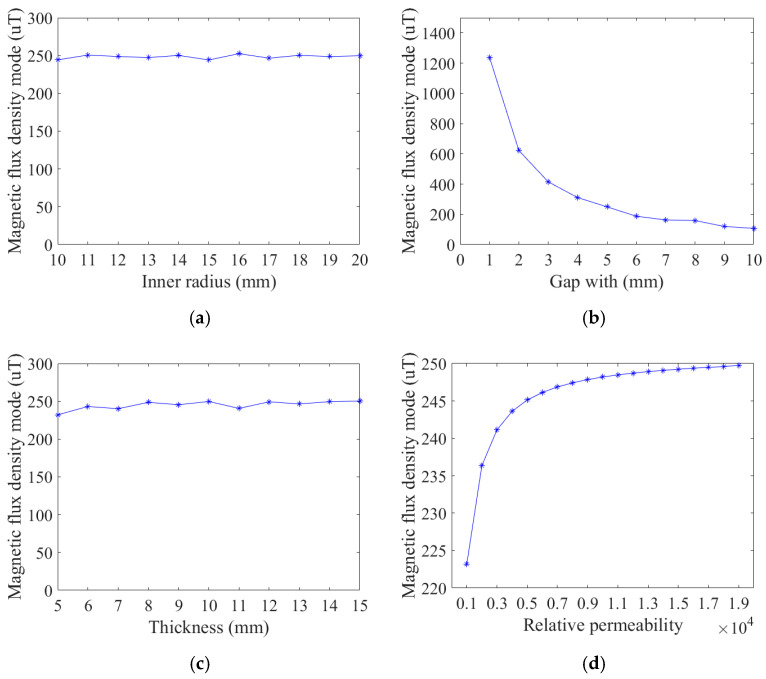
Simulation results of magnetic ring structure parameters: (**a**) inner radius simulation; (**b**) the gap width simulation; (**c**) the thickness simulation; and (**d**) the relative permeability simulation.

Next, based on the basic settings, the inner radius, thickness, relative permeability, and air gap width of the magnetic ring were carefully scanned and simulated, and the simulation results obtained are shown in Figure 5.

According to the simulation results in Figure 5, the relative magnetic permeability and air gap width of the magnetic ring are the main factors affecting the sensitivity of the TMR current sensor. The sensitivity of the sensor decreases with the increase in the air gap width and increases with the increase in the relative magnetic permeability of the magnetic ring. Moreover, when the relative magnetic permeability of the magnetic ring exceeds 3000, the rate of sensitivity increase slows down with the rise of the relative magnetic permeability of the magnetic ring.

According to the above simulation results, we set the structural parameters of the selected magnetic ring to an inner diameter of 20 mm and a thickness of 10 mm. Furthermore, to leave sufficient space for the TMR chip, the air gap width is set to 5 mm.

**Figure 6:**
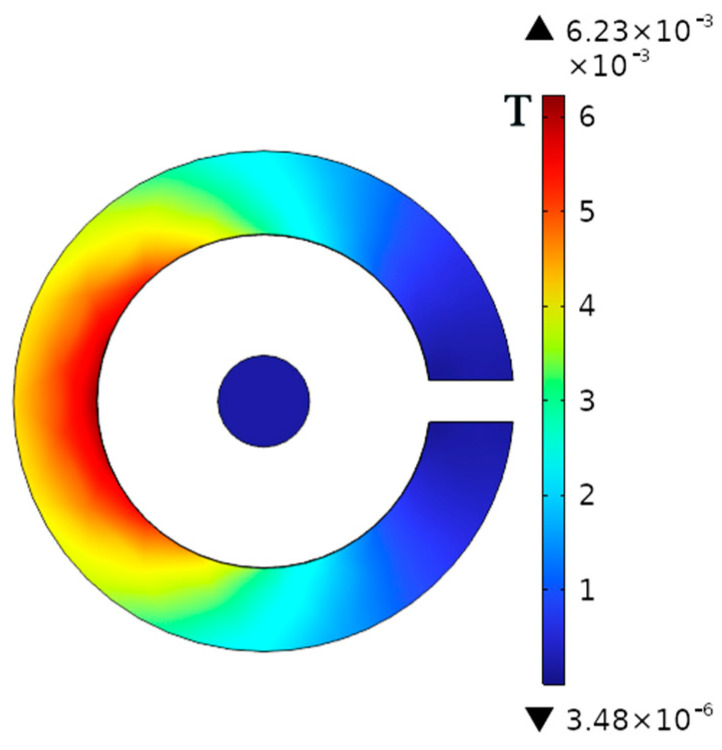
Magnetic flux density distribution when a magnetic ring shields an external magnetic field.

Subsequently, a magnetic shielding simulation was conducted on the magnetic ring with determined parameters. A stray magnetic field of 0.1 mT was applied along the y-axis, and the current in the wire was removed. The simulation results are illustrated in Figure 6.

The simulation results show that the magnetic field density at the center of the air gap of the magnetic ring is very small, only 3.6 μT, which is much less than the 0.1 mT of the external stray magnetic field. Therefore, the magnetic ring itself has the effect of shielding the magnetic field. To further enhance the sensor’s resistance to magnetic interference, the shielding housing part is designed next.

## 3. Design and Simulation of TMR Current Sensor Shielding Shell

### 3.1. Analysis of the Source of Magnetic Interference Error in TMR Current Sensor

The TMR current sensor is a high-sensitivity current sensor designed based on the current loop theorem. However, it is usually sensitive to magnetic field interference in measurements, resulting in inaccurate measurements, especially in low current measurements, and even in the inability to distinguish between target signals and stray signals.

External stray magnetic fields, including geomagnetic transverse fields, mobile communication, and discharge equipment, emit electromagnetic radiation during the measurement process, which affects the accuracy of current sensors.

In an ampere loop, when the angle *θ* is stored between the magnetic field *H* at a certain point in length dl and the tangent direction of the loop in the closed path, the integral result of the loop is as follows:

(8) $$\int_{dL}\vec{H}dl=\int_{dL}H\cos\theta dl=H\cos\theta dl$$

**Figure 7:**
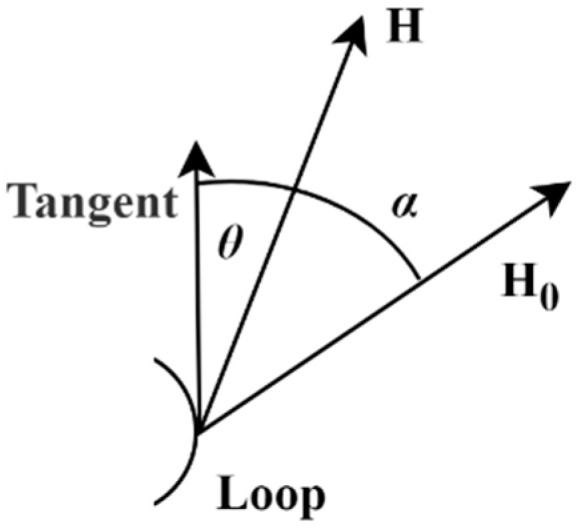
The principle of interference.

When the loop’s magnetic field is influenced by an external magnetic field *H*_0_, it combines with the magnetic field generated by the current-carrying wire to form a resultant magnetic field *H*_1_. The angle between this resultant magnetic field and the loop’s tangent direction is α, as illustrated in Figure 7, which depicts the interfering magnetic field. The integration error here is as follows:

(9) $$\Delta =\left|H_{0}\cos\alpha -H\cos\theta \right|dl$$

Consequently, the magnetic field at the air gap affecting the TMR element comprises both the magnetic ring induced by the wire current and the external stray magnetic field converged by the magnetic ring, leading to measurement errors in the current sensor.

### 3.2. Shielding Design and Parametric Simulation

Several complicated external radiation interferences are effectively prevented by the use of electromagnetic shielding technology, which also reduces the amount of electromagnetic noise that is dispersed spatially by reducing the transmission path of radiated electromagnetic noise. The shielding performance of shield materials is typically used to describe the shielding effectiveness of this material, which is commonly used to characterize the protection effectiveness of shielding materials. The formula is as follows:

(10) $$SE=20\mathrm{lg}\frac{H_{0}}{H_{S}}(\mathrm{dB})$$

*H*_0_ is the external stray magnetic field, and *H_s_* is the shielded magnetic field.

**Figure 8:**
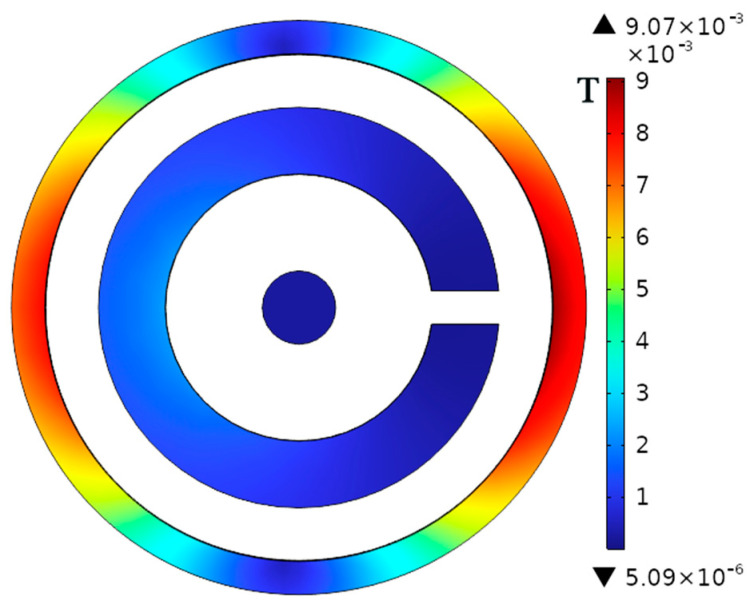
The shielding effect of the housing.

From Formula (10), it can be known that the magnetic ring shielding effectiveness in the previous text is 28 dB. On this basis, a 5-mm-thick annular shielding shell is added, and its material is also permalloy. Figure 8 shows the resulting magnetic flux density distribution. From the simulation results, it can be known that the shielding effectiveness at this time is 38.41 dB. Therefore, based on the original magnetic ring shielding, the external shielding shell can further enhance the magnetic shielding capability of the current sensor.

**Figure 9:**
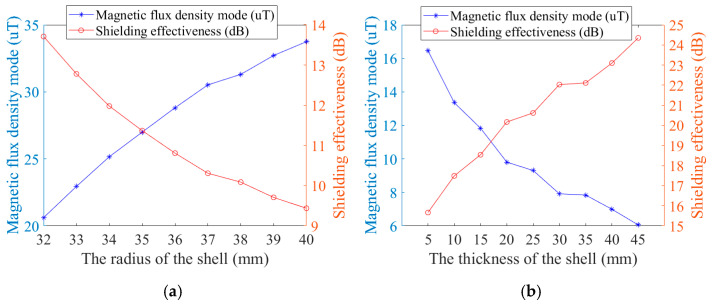
Shielded enclosure simulation results: (**a**) the simulation results for radius and (**b**) the simulation results for thickness.

Figure 9 presents the simulation outcomes following the scanning of the shield shell’s radius and thickness. Simulation results indicate that increasing the thickness of the shielding shell enhances its efficiency and the smaller the radius, the greater the shielding effect.

Ferromagnetic materials can be divided into two kinds: one is soft magnetic materials, which means that even if the material is under the action of a weak external magnetic field, the magnetic induction intensity B can reach a very high value, and at the same time has a very low coercivity, that is to say, it is easy to be magnetized, and it is easy to demagnetize the material, such a material is a soft magnetic material. It includes industrial silicon steel materials, permalloy, iron-nickel alloys, etc. Compared with soft magnetic materials, another ferromagnetic material we call hard magnetic materials is a material that is difficult to magnetize and is difficult to achieve demagnetization after being magnetized, which mainly includes carbon steel, tungsten steel, barium ferrite, etc., and its main application is the production of permanent magnets. The magnetic shields we design generally require materials with low coercivity and high initial permeability, so soft magnetic materials are more suitable.

**Figure 10:**
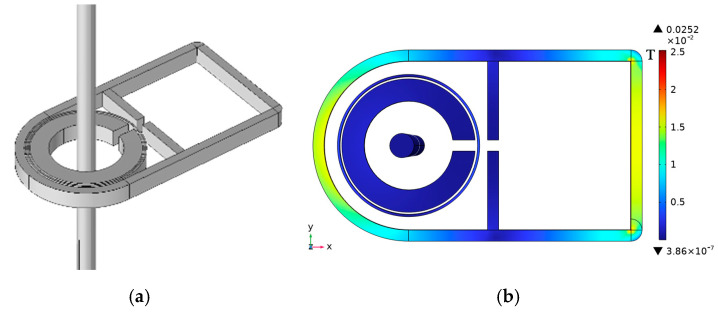
Shielded enclosure structure and flux density distribution: (**a**) schematic diagram of the modeling of the magnetic shield structure; (**b**) magnetic flux density distribution of a magnetically shielded structure.

To achieve better shielding efficiency, the circuit board and magnetic ring are assembled, and a 10 mm thick shielding shell is added on top of the single-layer annular shielding shell to tightly bond the inner shielding shell and magnetic ring. This structure is designed to be suitable for integration into circuit boards. Figure 10 shows a schematic diagram of its structure and magnetic flux density distribution. Similarly, dark and warm areas have higher magnetic flux density, while bright and cool areas have lower magnetic flux density. To enhance the shielding effect, the outer shell material is set to a microcrystalline alloy with a relative magnetic permeability of 600,000, and the inner layer is composed of aluminum. This is because aluminum has high conductivity and can shield some high-frequency electromagnetic fields, further enhancing the anti-interference ability of the sensor. Fill the remaining gaps with silicone gel. The shielding effect is 40.4 dB, which is 44.3% higher than the single magnetic ring structure.

## 4. Simulation and Experimental Results

### 4.1. Simulation of Shielding Effect of Shielding Shell

**Figure 11:**
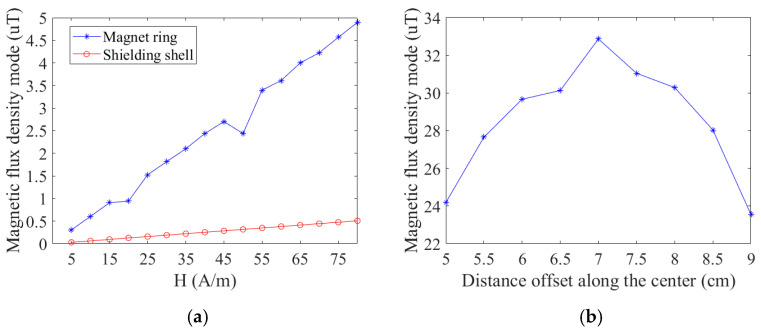
Shielding effectiveness simulation results: (**a**) simulation results of shielding effectiveness of magnetic shielding structure; (**b**) simulation results of the protective circuit effect of the magnetic shielding structure.

The shielding effectiveness test is carried out on the designed shielding enclosure. The figure illustrates the comparison between a single magnetic ring and a shielded shell structure under an increased external magnetic field. The sensor with a single magnetic ring structure exhibits a greater impact than the sensor with an externally shielded shell as the external magnetic field strengthens. When current flows through the wire in an external stray magnetic field of 0.1 mT, the measurement error for the shielded shell sensor is 9.5%, while the single magnetic ring sensor has a 20% error. The package minimally affects the current sensor’s sensitivity, with an error of less than 1% compared to the single magnetic ring. The shielded package enhances the accuracy of TMR current sensors in external stray magnetic fields, achieving 2.1 times the precision of traditional single-ring sensors. Figure 11b illustrates that the magnetic flux density modulus along the circuit board axis and air gap demonstrates effective shielding of external magnetic fields, with a uniform distribution indicated by a difference of no more than 10 µT between the center and edge.

### 4.2. Experimental Testing of Shielded Housings

**Figure 12:**
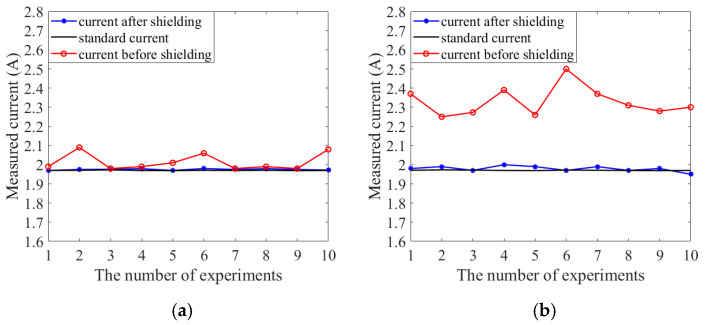
Actual test results of the shielding cover: (**a**) test results without interference; (**b**) test results in the presence of interference.

Next, the designed shielding structure was experimentally tested. The TMR sensor chip adopts the TMR2901 of China JiangsuMultiDimension Technology Co., Ltd. (Suzhou, China). The test current is generated by the voltage source and resistance, and the current is collected by a digital multimeter as the standard current value (theoretical value 1.97 A). The TMR current sensor without the shielded structure and the TMR current sensor with the shielded structure were tested separately, and the energized Helmholtz coil was used as the interference source (the axial magnetic field is approximately 100 μT). Figure 11 shows the test results. Figure 12a shows the test results without interference, and Figure 12b shows the measurement results with interference. From the test results, it can be seen that when there is magnetic field interference from the outside world, the TMR current sensor under the shielded structure is almost not affected by the outside world, while the measurement error of the TMR current sensor without the shielded shell reaches 19.49%. Therefore, the shield structure can effectively reduce the measurement error of the TMR current sensor. The shielding effect of the shielding structure is clear. This is consistent with the simulation results.

## 5. Discussion and Conclusions

This paper examines the benefits of closed-loop TMR current sensors compared to open-loop TMR current sensors within a magnetic ring structure, highlighting their superior temperature characteristics and linearity. The simulation is designed based on the closed-loop TMR current sensor structure. Scanning simulations are performed on key magnetic ring parameters, radius, thickness, material, and void width. The relationship between the sensitivity of the TMR current sensor and the four is obtained, and the degaussing ability of the feedback coil is verified by simulation. Based on the design of the maximum range of 100 A, the geometric parameters are determined. The analysis indicates that external stray magnetic fields in the ampere loop can interfere with the TMR element, necessitating the design of a shielding shell to block this radiation. The influence of the thickness and radius of the shield on the shielding effectiveness was analyzed, and the shield package structure that could be loaded with circuit boards was designed. With a stray magnetic field of 0.1 mT along the sensitive axis, the shielding efficiency surpasses that of a single magnetic ring by 44.3%, and the measurement accuracy is enhanced by 2.1 times compared to the traditional structure. In the experimental test, the measurement accuracy of the sensor with the shielded case is significantly improved compared with the unshielded TMR current sensor. The shielding structure designed in this paper is more suitable for the environment of geomagnetic fields and low-frequency magnetic fields and is suitable for the detection of power frequency current in the power grid, which provides a strong theoretical and experimental basis for the design of the TMR current sensor.

## Data Availability

The data that support the findings of this study are available from the corresponding author upon reasonable request.
